# Bacterial outer membrane vesicles as drug delivery carrier for photodynamic anticancer therapy

**DOI:** 10.3389/fchem.2023.1284292

**Published:** 2023-10-17

**Authors:** Yuan Jiang, ZunZhen Zhou, Chongzhi Liu, Limei Wang, Chun Li

**Affiliations:** ^1^ Clinical Medical College and the First Affiliated Hospital of Chengdu Medical College, Chengdu, Sichuan, China; ^2^ Department of Rehabilitation Medicine, The Third Affiliated Hospital of Chengdu Medical College, Chengdu, Sichuan, China; ^3^ Department of Rehabilitation Medicine, Mianyang Central Hospital, School of Medicine, University of Electronic Science and Technology of China, Mianyang, China

**Keywords:** photodynamic therapy, photosensitizers, drug delivery carrier, nanoparticles, outer membrane vesicles

## Abstract

Photodynamic Therapy (PDT) is an effective tumor treatment strategy that not only induces photocytotoxicity to kill tumor cells directly but also activates the immune system in the body to generate tumor-specific immunity, preventing cancer metastasis and recurrence. However, some limitations of PDT limit the therapeutic efficacy in deep tumors. Previous studies have used different types of nanoparticles (NPs) as drug carriers of photosensitizers (PSs) to overcome the shortcomings of PDT and improve therapeutic efficacy. Among them, bacterial outer membrane vesicles (OMVs) have natural advantages as carriers for PS delivery. In addition to the targeted delivery of PSs into tumor cells, their unique immunogenicity helps them to serve as immune adjuvants to enhance the PDT-induced immune effect, providing new ideas for photodynamic anticancer therapy. Therefore, in this review, we will introduce the biogenesis and anticancer functions of OMVs and the research on them as drug delivery carriers in PDT. Finally, we also discuss the challenges and prospects of OMVs as a versatile drug delivery carrier for photodynamic anticancer therapy.

## Introduction

Photodynamic Therapy (PDT) is a photochemical treatment triggered by light irradiation, and it has played a good role in the clinical treatment of tumors, precancerous lesions, infectious diseases, and other diseases as an alternative therapy in recent years. The principle of PDT is based on the excited photosensitizers (PSs) to produce reactive oxygen species (ROS) upon light irradiation at a specific wavelength, resulting in photocytotoxicity to damage the target tissue or cells (Kwiatkowski et al., 2018). Low-toxicity PSs, non-invasive light irradiation, and oxygen are the three core elements of PDT, so many scholars suggested that PDT is a promising treatment modality for cancer with better therapeutic effects and fewer invasiveness and side effects than conventional therapies (e.g., surgery, chemotherapy, and radiotherapy). PDT combined with endoscopic and fiberoptic light delivery techniques can expand the cancer treatment area, which may be inaccessible by surgery. The administration of light irradiation also exhibits high spatiotemporal control, reducing off-target toxicities (Overchuk et al., 2023). With the continuous deepening of research on PDT, researchers have found that in addition to killing tumor cells, PDT can also affect the body’s immune function and activate the immune system, resulting in tumor-specific immunity and preventing cancer metastasis and recurrence (Donohoe et al., 2019; Ji et al., 2022). In addition, the rare reports of drug resistance also make scholars believe that PDT will be a promising treatment modality for cancer (Lo et al., 2023). Some factors can affect the efficiency of PDT and its anti-tumor immune response, particularly the limitations of the inherent nature of PSs and light and complex tumor microenvironment (TME) (Hamblin and Abrahamse, 2021). First, most PSs are lipid-soluble drugs with poor stability, which need to be dissolved in organic solvents, making them unsuitable for intravenous administration. Second, although PSs will gather in tumor tissue, their low tumor selectivity and poor pharmacokinetics also cause photo-damage to normal tissue adjacent to tumor tissue and skin tissue (Kwiatkowski et al., 2018). Third, the excitation light of most existing PSs is short-wavelength ultraviolet-visible light (400–700 nm), and the strong absorption and scattering of light within this wavelength range by skin tissue make poor penetration depth, leading to PSs-induced PDT hard to achieve effective treatment of solid tumors in deep tissues. Although long-wavelength NIR light (700–1,100 nm) is used to activate some PSs with greater tissue penetration, the effect of NIR light on PDT in deeper tissues remains limited (Gao et al., 2022a). Fourth, solid tumors’ rapid growth with an underdeveloped vascular network causes a hypoxic state in TME that will limit the ROS level produced by PDT (Du et al., 2022; Li et al., 2023), and high concentrations of glutathione (GSH) in cancer cells can also consume ROS, leading to the inefficiency of PDT (Xiong et al., 2021; Wang et al., 2022b). Therefore, scholars have devoted themselves to seeking efficient and feasible methods to solve the above problems to promote the further development of PDT.

With the introduction of nanomaterials in PDT, based on their feature of the nanoscale structure, variable morphology, low biological toxicity, good biocompatibility, and easy modification, leading different types of nanoparticles as drug carriers of PSs provided more possibilities to overcome defects of PDT (Younus et al., 2023). For example, up-conversion nanoparticles (UCNPs) and persistent luminescence nanoparticles (PLNPs) can be used as drug delivery carriers to overcome the poor penetration depth of excitation light sources (Qiu et al., 2018; Fritzen et al., 2020). Gold nanoparticles (GNPs) as drug delivery carriers of PSs can generate additional photothermal effects under near-infrared (NIR) irradiation to improve the poor anti-cancer efficacy of PDT caused by hypoxia (Kim and Lee, 2018). Iron oxide nanoparticles (IONPs) can load PSs and then achieve MRI imaging guidance of tumors before or after PDT (Rajkumar and Prabaharan, 2017). Mesoporous silica nanoparticles (MSNs) have a large surface area and pore volume that can encapsulate PSs, small interfering RNA (siRNA), and chemotherapy drugs/prodrugs to achieve synergistic anti-tumor effects. Their surface can also be modified with functional groups such as folic acid (FA) and aptamers to provide the ability to target tumors and improve the distribution of PSs in the tumors (Prieto-Montero et al., 2023). Organic nanomaterials can be self-assembled into amphiphilic compounds, forming nanospheres or micelles to carry PSs and other drug formulations (Park et al., 2021). In addition, some modified nanomaterials can be responsive to the physiological characteristics of the tumor (e.g., enzyme and pH) and their microenvironment (e.g., hypoxia, GSH, hydrogen sulfide, H_2_O_2_, and H^+^) or externally applied physical stimuli (e.g., light, heat, ultrasound) to realize controlled release of PSs at specific sites (Zheng et al., 2020). However, scholars have raised concerns about biological toxicity, biodegradability, safety, immunogenicity, and tolerance of these nanomaterials after intravenous administration (Tong et al., 2022). These uncertainties limit the vast majority of research on the nanomaterials-based-delivery system of PSs to preclinical studies, which is a big challenge for them in achieving clinical conversion.

Extracellular vesicles (EVs) are nanosized lipid-bound vesicles with a size range of 30–150 nm in diameter released from cells. EVs have a lipid bilayer membrane that contributes to carrying exogenous anticancer drugs or therapeutic agents. In contrast to conventional synthetic nanomaterials, EVs have some advantages, such as low toxicity, low immunogenicity, high biocompatibility, and good stability in blood circulation (Elsharkasy et al., 2020; Meng et al., 2020). EVs also possess intrinsic cell-targeting properties and tissue-homing capabilities that liposomes do not have (Herrmann et al., 2021). In addition, through chemical methods, EVs can be gifted tumor-targeting ability by directly conjugating with targeting ligands (e.g., aptamers, antibodies, and peptides) (Jiang et al., 2022). Therefore, EVs as a natural drug delivery system have opened new frontiers for modern drug delivery, especially the delivery of PSs to the tumor tissues, representing a promising novel strategy of photodynamic anticancer therapy (Kusuzaki et al., 2017). More and more studies have confirmed that bacteria can secrete EVs similar to human cells, outer membrane vesicles (OMVs) (Sartorio et al., 2021; Liu et al., 2022). OMVs take part in many physiological processes of their parental bacteria, including nutrient acquisition, antibacterial defense, horizontal gene transfer, biofilm formation, virulence factor delivery, and intracellular/extracellular communication, and their biological function plays an essential role in the survival of the bacteria (Toyofuku et al., 2019). The application of OMVs as versatile tools for therapeutic approaches has attracted the attention of scholars because of their inherent ability to elicit an immune response in the body, especially in cancer immunotherapy (Zingl et al., 2021). At first, OMVs can be immunologic adjuvant or tumor vaccines for activating the body’s immune system in antitumor therapy (Chen et al., 2022; Hosseini-Giv et al., 2022; Long et al., 2022). Previous studies have confirmed that OMVs can specifically target tumor tissue and accumulate in tumor tissue after intravenous administration. OMVs cannot replicate and have higher safety than bacteria, indicating that OMVs have a natural advantage as a delivery carrier for antitumor drugs (Gujrati et al., 2014; Kim et al., 2017; Wang et al., 2019). Recent studies used OMVs as drug delivery carriers to enhance the PDT-induced immune effects and even achieve combinational photodynamic/chemo-/immunotherapy in tumor-bearing mice by simultaneously delivering chemotherapy drugs (Li et al., 2022; Zhuang et al., 2022). These works provide a novel idea for photodynamic anticancer therapy by developing OMVs-based drug delivery carriers.

Therefore, in this review, we will introduce the biogenesis and anticancer functions of OMVs and the research on them as drug delivery carriers in PDT. Finally, we also discuss the challenges and prospects of OMVs as a drug delivery carrier for photodynamic anticancer therapy.

## Biogenesis of OMVS

**FIGURE 1:**
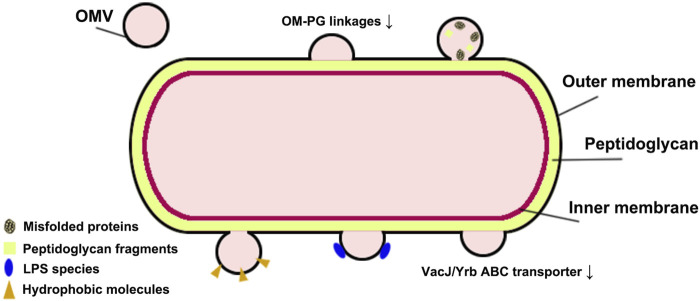
Changes in OMV biogenesis.

OMVs are non-living spherical double-layer vesicle structures released by Gram-negative bacteria and some Gram-positive bacteria, mainly composed of the peptidoglycan layer and periplasm, containing bacterial bioactive proteins, lipids, nucleic acids, and metabolites (Jahromi and Fuhrmann, 2021; Ozkocak et al., 2022). Gram-negative and Gram-positive bacteria have obvious differences in cell structure, leading to different biogenesis processes and membrane components between their OMVs. Gram-negative bacteria have an outer membrane (OM) structure caused by the thin peptidoglycan membrane in the periplasm separating the OM from the inner membrane. OM is composed mainly of lipopolysaccharide (LPS), while the inner membrane riched phospholipid. OMVs mainly originate from the budding of the OM and explosive cell lysis (Zingl et al., 2021). The budding of OM will produce the classic OMVs after cell envelope disturbance caused by the following changes, as shown as Figure 1 (Kulp and Kuehn, 2010; Roier et al., 2016; Sartorio et al., 2021). a) the outer membrane-peptidoglycan protein (OM-PG) linkages are directly lost or by the movement of the linking protein; b) the local accumulation of peptidoglycan fragments or misfolded proteins in the periplasm; c) the accumulation of phospholipids in the outer leaflet of the OM via the downregulation of VacJ/Yrb ABC transporter; d) the local enrichment of LPS species with anionic charges; e) the hydrophobic molecules insert into the OM. These OMVs are characterized as having no cytoplasmic components as their content because the inner membrane remains intact (Wang et al., 2022a). Other types of OMVs of Gram-negative bacteria include outer-inner membrane vesicles (OIMVs) and explosive outer membrane vesicles (EOMVs) caused by explosive cell lysis (Pérez-Cruz et al., 2015; Delgado et al., 2019; Mandal et al., 2021). OIMV are the results of the vesicles containing cytoplasmic contents (such as DNA), which originated from the inner membrane protruding into the periplasm after endolysin degrading the peptidoglycan cell wall, are squeezed out from the cell surface with surrounding OM. EOMVs, randomly encapsulated released DNA vesicles, are formed by the re-circulation, aggregation, and self-assembly of the shattered membrane fragments induced by DNA damage-induced cell lysis. Gram-positive bacteria have a thick peptidoglycan layer outside but lack OM structure, and the released vesicles are derived from the cytoplasmic membrane (CM) (Toyofuku et al., 2023). Existing research suggests that endolysin-triggered “bubbling cell death” in Gram-positive bacteria forms the cytoplasmic membrane vesicles (CMVs), which can contain membrane and cytoplasmic components. Some studies have also observed specialized types of MVs with tube-shaped mechanical structures, which may be derived from the tube-like protrusions of the cytoplasmic membrane of Gram-positive bacteria or the tube-like outer membrane of Gram-negative bacteria. Thus, the biogenesis processes of OMVs and CMVs differ, indicating that they differ in origin, components, and function. Tian and others summarized the differences between OMVs and CMVs in their published review (see reference Tian et al., 2023). The production of OMVs is a spontaneous bacterial behavior in response to external stress. So the biogenesis processes of OMVs would be affected by temperature, growth environment, Quorum sensing (QS), bacterial growth stage, and hereditary factors, and even their contents will change accordingly, affecting their biological functions.

## Antitumor action of OMVS

**FIGURE 2:**
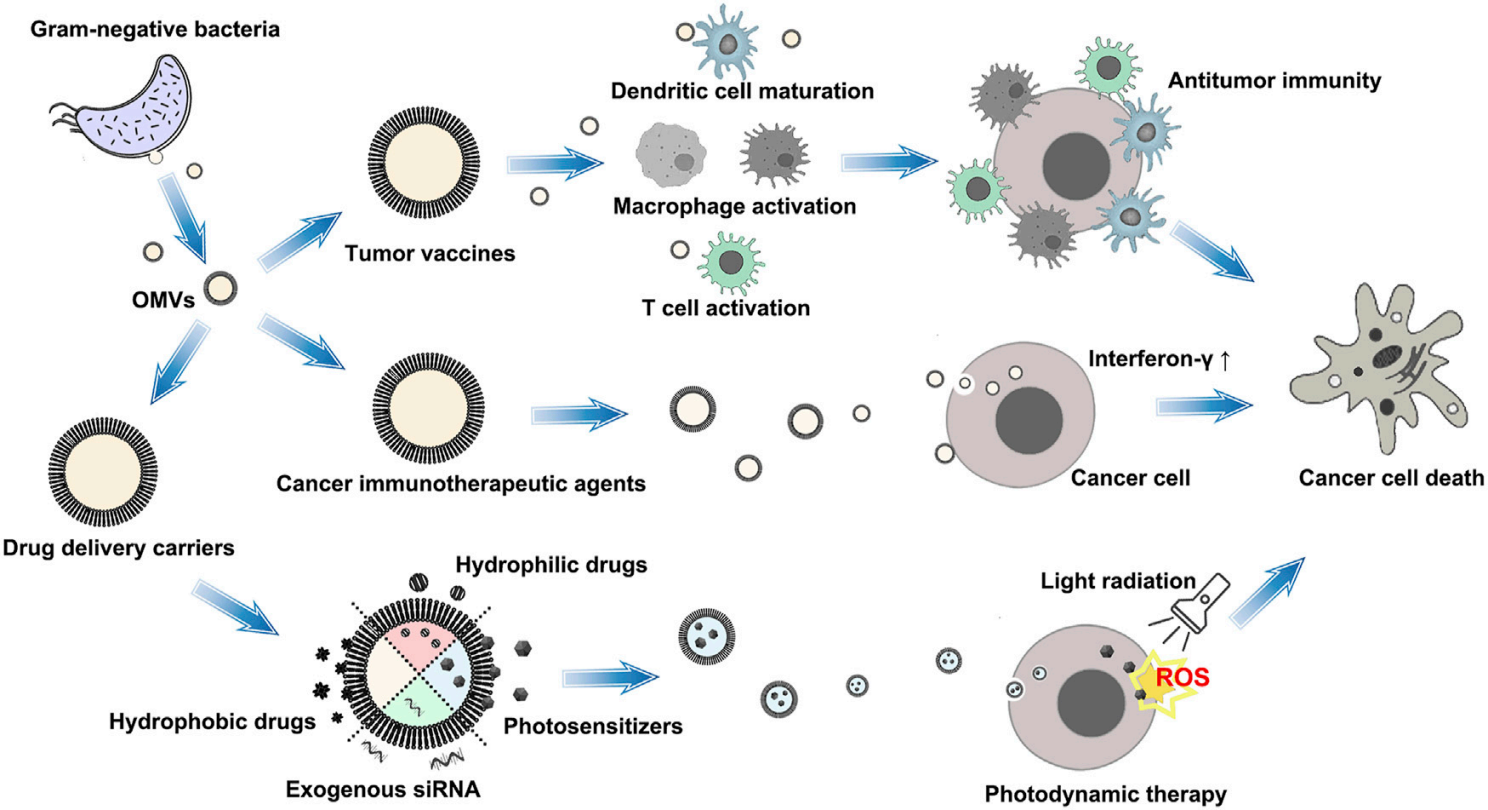
Schematic illustration of the application of OMVs’ antitumor action. The therapeutic strategy of OMVs mainly includes tumor vaccines, cancer immunotherapeutic agents, and antitumor agent delivery carriers.

OMVs are derived from the OM and have the outer membrane proteins (OMP), LPS, lipoproteins (LPP), and peptidoglycan, which belong to pathogen-related molecular patterns (PAMPs). PAMPs can bind to pathogen recognition receptors on immune cells, activating the innate immune response. OMVs can passively target the tumor via the enhanced permeability and retention (EPR) effect (Suri et al., 2022). With these characteristics, OMVs have demonstrated unique advantages in antitumor therapy. To date, the application of OMVs’ antitumor action mainly includes tumor vaccines, cancer immunotherapeutic agents, and antitumor agent delivery carriers, as shown as Figure 2).

### OMVs as tumor vaccines

Cancer immunotherapy activates the host immune system to attack and eliminate tumor cells and has become an important treatment strategy, including vaccines, cytokines, immune checkpoint inhibitors, etc. Among them, tumor vaccines based on their safety and reliability have also been a hot research field in recent years, mainly utilizing tumor-related antigens to overcome immune suppression caused by tumors, leading to enhancing immunogenicity and activating the immune system. OMVs possess intrinsic immunogenicity, and their tumor antigens can further stimulate the body’s immune response, which is beneficial for their development and application as tumor vaccines (Gao et al., 2022b). OMVs-based anti-tumor vaccines are mainly developed from functionally modified by genetic engineering of bacteria to cause a foreign protein to be expressed in the vesicle lumen or on its membrane surface (Wang et al., 2022b). For example, some antigens can be fused with the protein from the OMVs, such as cytolysinA (ClyA), hemoglobin protein (Hbp), and OMP, to form chimeric protein on the OMV membrane. Cheng and others used ClyA to fuse the catcher protein SpC/SnC, and the specific antigen protein binds to the tag protein SpT/SnT by a peptide bond, leading to various tumor antigens can be rapidly and simultaneously displayed on the OMVs surface via the binding to protein tags. This OMV-based vaccine platform elicits a specific anti-tumor immune response via specifically presenting antigens onto the OMV surface to inhibit metastasis and growth of tumors and induce long-term immune memory (Cheng et al., 2021). Huang and others used genetic recombination technology to fuse the thioredoxin (Trx) gene and the full-length mouse BFGF gene into the pThioHisA plasmid. The plasmids were transformed into *E. coli* DH5α competent cells to induce Trx-BFGF fusion protein expression and localized in the periplasmic space under the guidance of the Trx protein and loaded onto OMVs to form BFGF-modified OMVs (BFGF-OMVs). They found that BFGF-OMVs is a novel therapeutic tumor vaccine that can induce the body to produce antiangiogenesis autoantibodies to inhibit tumor angiogenesis through active immunization. The persistent anti-BFGF autoantibodies can be observed in tumor-bearing mice after only three times BFGF-OMVs intervention, which exerts tumor suppression effects (Huang et al., 2020).

### OMVs as cancer immunotherapeutic agents

OMVs have not only the immune regulatory ability but also have antitumor effects and can be used as cancer immunotherapeutic agents. Kim and others investigated the potential of OMVs as an immunotherapeutic agent for cancer. At first, They harvested the OMVs derived from lipid A acyltransferase (msbB) mutant *E. coli*, whose gene encoding the lipid component of lipopolysaccharide (lipid A acyltransferase) had been inactivated to avoid possible adverse effects by endotoxin lipopolysaccharide. After being injected intravenously via the tail vein, OMVs can target tumors and accumulate in tumor tissue without any targeted modification. OMVs induced long-term antitumor immune responses in tumor-bearing mice models and eradicated tumors by producing IFN-γ within the tumor microenvironment (Kim et al., 2017).

### OMVs as anti-tumor agent delivery carriers

The double-layer membrane structure, tumor-targeting ability, gene or membrane modifiability, and the ability of long-distance delivery of active molecules of OMVs make them promising candidates as new types of antitumor agent delivery carriers. In particular, OMVs from certain bacteria can disrupt the mucosal and epithelial barrier integrity to facilitate OMVs to pass biological barriers (Jahromi and Fuhrmann, 2021). Doxorubicin (DOX) is a broad-spectrum antineoplastic agent, but it non-specifically targets all kinds of cells, resulting in adverse effects such as cardiotoxicity, myelosuppression, and immunosuppression. To alleviate the cytotoxicity of DOX to normal tissues, Kuerban and others utilized OMVs from attenuated *Klebsiella* pneumonia as the delivery carriers to load DOX. In their study, DOX and OMVs were gently mixed in PBS and then incubated at 37°C to form the DOX-loaded OMVs (DOX-OMV). DOX was efficiently transported into non-small-cell lung cancer cells by OMVs, leading to intensive cytotoxic effects and cell apoptosis. While OMVs elicit appropriate immune responses to enhance the antitumor effect of DOX with no evident toxic side effects and adverse reactions in tumor-bearing nude mice (Kuerban et al., 2020). Gujrati and others first demonstrated the possibility of loading siRNA into the OMVs to achieve tumor-targeted siRNA delivery. They employed the OMVs derived from the msbB mutant *E. coli* transformed with pGEX4T1-ClyA-affibody construct as the delivery carriers. These OMVs display a human epidermal growth factor receptor 2 (HER2)-specific affibody in the membrane as a targeting ligand, named AffiHER2OMVs. The TAMRA-labeled siRNA targeting kinesin spindle protein (KSP) as a model therapeutic agent was loaded into AffiHER2OMVs using electroporation. The siRNA-loaded OMVs can induce targeted gene silencing and significant inhibition of cell proliferation in HER2-overexpressing cell lines and tumor growth regression in tumor animal models rather than elicit their antitumor effects by overstimulating inflammatory or immunological pathways (Gujrati et al., 2014). Chen and others mixed *E. coli*-derived OMVs and AuNPs in a homogenizer to form stable complex Au-OMVs, indicating that OMVs can load nanoparticles on their surface. They found that Au-OMVs possess the ability to produce immuno-modulatory and radiosensitizing effects. Au-OMVs induce high intracellular ROS, chemotaxis of macrophages, and high levels of TNF- α, leading to a specific cytotoxic effect on glioma cells and reducing radiotherapy dosage in tumor-bearing mice (Chen et al., 2021). Shi and others selected the *E. coli*-derived OMVs to encapsulate 5-fluorouracil (5-FU)-loaded MSNs by high-pressure co-extrusion. OMVs as delivery carriers can improve the stability of the 5-FU-loaded MSNs, reduce the leakage of 5-FU, and enhance the accumulation of 5-FU-loaded MSNs in colon cancer cells that contribute to MSNs accurately releasing 5-FU in the lesion (Shi et al., 2020). Lately, Cui and others designed an efficient miRNA nano-delivery system for tumor gene therapy based on the PD1 displayed OMVs to encapsulate zeolitic imidazolate framework-8 (ZIF-8) containing miR-34a. The engineered OMVs exhibited high miRNA delivery efficiency, tumor targeting, immune activation, and checkpoint inhibition, representing promising biomimetic nano-delivery carriers for the intracellular delivery of miRNA to enhance tumor therapeutic efficacy (Cui et al., 2023).

The above research indicates that OMVs have shown multiple potential applications and research value in tumor therapy. Therefore, we wonder how to utilize the advantages of OMVs in tumor treatment to bring some new thinking to the research of photodynamic anticancer therapy.

## OMVS in photodynamic anticancer therapy

In recent years, the stimulating effect of OMVs in tumor immune response and their ability to act as drug carriers have attracted scholars’ attention and have inspired their innovative attempts to apply OMVs to photodynamic anticancer therapy, as shown as Table 1. Zhang and others used attenuated *Salmonella*-derived OMVs as versatile drug carriers to overcome hypoxia in tumor tissue and improve PDT-induced immune response. They first synthesized a nano-complex (CAT-Ce6) from hydrophilic catalase (CAT) and hydrophobic PS (Chlorin e6, Ce6) via self-assembly. CAT-Ce6, as an amphiphilic complex, can avoid the aggregation of Ce6 in the aqueous solution, improving biocompatibility and distribution of Ce6 in tumor tissues. CAT-Ce6 has the catalytic activity of CAT, which can decompose H_2_O_2_ in tumor tissues to generate oxygen to improve the hypoxic state, thereby enhancing the photodynamic killing effect on tumors. Then they modified the programmed death-ligand 1 antibody (aPDL1) on the surface of OMVs by extrusion, forming OMV-aPDL1 as the delivery carriers for CAT-Ce6. After transferring CAT-Ce6 to tumor tissues, OMV-aPDL1 can activate anti-tumor immune responses and dendritic cells (DCs) to induce CD8 T cells to move to tumor tissues. While aPDL1 can blockade PD-1 to relieve the immunosuppressive effect, leading to eliminating tumor immune escape. Their experimental results prove that CAT-Ce6@OMV-aPDL1 can achieve the synergy of oxygenerated PDT and immunity for improving the efficiency of cancer treatment and even induced Immunological memory effect in mice receiving treatment twice (Zhang et al., 2022). Peng and others utilized OMV’s ability to penetrate the stratum corneum (SC) and designed OMV-based versatile delivery nanoplatforms for skin melanoma treatment, named I-P-OMVs. They harvest the attenuated OMVs derived from TRAIL gene-transformed *E. coli* and then modified αvβ3 integrin targeting peptide (RGP) on the surface of OMVs and load the indocyanine green (ICG) by simply co-incubation to form I-P-OMVs. Based on their design features, I-P-OMVs can penetrate SC via follicle routes and target skin melanoma through a specific binding with αvβ3 integrin at the surface of melanoma cells. The ICG loaded by I-P-OMVs can induce photodynamic/photothermal effects in melanoma spheroids under NIR irritation, leading to a high level of ROS and hyperthermia to kill melanoma cells. The photothermal effects can destroy OMVs to release TRAIL, which binds to death receptors in melanoma cells’ surface, activating the apoptosis in residual melanoma cells. These synergistic therapeutic effects can prevent cell proliferation and invasion of melanoma cells by interfering with the relevant genes and proteins and delaying the progression, relapse, and metastasis of melanoma with good biosafety in the mice model, indicating that I-P-OMVs represent a feasible OMVs-based versatile nano-platform with great potential in photo-treatment of melanoma (Peng et al., 2020). Li and others developed a macrophage-mediated OMV-based delivery carrier (OMVs@M) to transmit Ce6 and the chemotherapeutic drug doxorubicin (DOX) into triple-negative breast tumors, providing combinational photodynamic/chemo-/immunotherapy to eradicate and prevent tumor metastasis. In their research, *E. coli* -derived OMVs were uptaken by macrophages to form OMVs@M as delivery carriers, which can enhance OMVs’ tumor-targeting ability and safety via macrophage-mediated delivery, and OMVs can lead to M2-to-M1 polarization of macrophages and activate pyroptosis to improve antitumor immunity. Based on this bioengineering strategy, OMVs containing Ce6/DOX were uptaken by macrophages to form a versatile therapeutic platform (DOX/Ce6-OMVs@M) for laser-triggered photodynamic effect and synergic antitumor therapy (Li et al., 2022). Zhuang and others constructed versatile *E.coli* OMV-based hybrid nanovesicles with phytochemical features to enhance photodynamic effects-promoted immunotherapy. Interestingly, they chose the plant-derived thylakoid membranes (Tk) instead of conventional PSs because these special membranes contain various enzymes and photosystems (photosensitive chlorophyll) that can induce efficient photodynamic effects. OMVs are fused with thylakoid membranes by co-extrusion to form bacteria-plant hybrid vesicles (BPNs). BPNs can target tumor tissues, stimulate the immune response, and even generate ROS under 660 nm light irradiation, indicating that BPNs integrated the immune-modulatory functions of OMVs as cancer vaccines with the photodynamic effects of Tk as PS. After only one light irradiation, BPNs can eliminate tumor growth and prevent tumor metastasis without any evident side effects in colon cancer CT26 tumor xenografted mice, suggesting that phytochemical-engineered OMVs represent a novel versatile membrane-based hybrid system for highly efficient tumor treatment (Zhuang et al., 2022). The above-presented works demonstrate that OMVs could be the ideal candidates as drug delivery carriers in photodynamic anticancer therapy and combine with other strategies for synergic antitumor therapy, indicating that OMVs may be an efficient and feasible method to solve the shortcomings of PDT and will promote the further development of PDT.

**TABLE 1:** Literature examples of OMVs as drug delivery carrier in photodynamic anticancer therapy.

Bacterial strain	Tumor type	Type of model	Loaded PS	Mode of action	References
*Salmonella*	Breast cancer	4T1 cell	Ce6	1) Solve the hydrophobicity problem of Ce6	Zhang et al. (2022)
		Mice		2) Activate anti-tumor immune responses	
*E*. *coli*	Melanoma	B16F10 cell	ICG	1) Skin penetration	Peng et al. (2020)
		Mice		2) Infiltrate and accumulate in tumor spheroid	
				3) Load and release TRAIL protein	
*E*. *coli*	Breast cancer	4T1 cell	Ce6	1) Activate macrophage to M1-like phenotype	Li et al. (2022)
		Mice		2) Induce pyroptosis in tumor cells	
				3) Load and release DOX.	
*E*. *coli*	Colon cancer	CT26 cell	Photosensitive chlorophyll	1) Target and accumulate in tumor tissues	Zhuang et al. (2022)
		Mice		2) Activate anti-tumor immune responses	

## Challenges of OMV-based drug deliver carriers

Of course, we also realize that published studies of OMVs as drug delivery carriers in photodynamic anticancer therapy are still limited. This situation can be attributed to OMVs having many challenges in their preparation and application.

### Safety

Although OMVs can not replicate like their parent bacteria and cause disease by themselves, they have bioactive proteins, lipids, nucleic acids, virulence factors, and metabolites from their parent bacteria. They may disrupt the microenvironment of target organs in complex human environments, leading to unexpected complications. For example, OMVs’ immunogenicity can activate the body’s immune response and cause immune storms, leading to adverse reactions and death (Matías et al., 2020). Qing and others observed that healthy Balb/c mice did not tolerate the OMVs treatments well after giving single-dose or multiple-dose intravenous (i.v.) injection regimes of the OMVs, and they speculated that the died mice died of some systemic inflammatory response (Qing et al., 2020). Hence, safety is the first challenge of OMVs as drug delivery carriers. How to attenuate the toxicity of OMVs with decreased levels of LPS is essential to OMV application (Li et al., 2020). However, using the detergents, genetic engineering, and physical or chemical methods to attenuate the toxicity may lead to the loss of bacterial antigens and lipoproteins and a decrease in the OMVs’ inherent immunogenicity, leading to OMVs will lose the immunoadjuvant effect (van de Waterbeemd et al., 2010; Li et al., 2020).

### Low yield

OMVs are naturally released from bacteria but in low quantities. Temperature, stress, low temperature, nutrient deficiency, antibiotics, and phages may increase OMV production during culture (Schwechheimer and Kuehn, 2015). But the large-scale production of OMVs still faces some difficulties and cannot meet the current clinical application standards. Some studies have shown that detergents (e.g., deoxycholate or sodium dodecyl sulfate) can stimulate bacteria to achieve higher production of OMVs but can cause unexpected changes in the properties of OMVs. Other methods have been reported to increase the production of OMVs, such as physical (e.g., sonication) or chemical (e.g., ethylenediaminetetraacetic acid, EDTA) treatment and genetic modifications. But scholars are also concerned that these methods could affect the size, stability, and composition of OMVs, resulting in the loss of the desired activity of OMVs (Naskar et al., 2021).

### Heterogeneity

The biogenesis processes of OMVs would be affected by bacterial strains, growth conditions, and stages of bacterial growth, leading to the heterogeneity between bacterial strains and species. Bitto and others compared the OMVs produced by *S. aureus*, *P. aeruginosa*, and *H. pylori*, and they suggested that the production, size distribution, and protein cargo quantity of OMVs vary within and between bacterial species, as shown as Table 2 (Bitto et al., 2021). Take protein content as an example, the amount of OMP/periplasmic proteins packaged into *E. coli*-derived OMVs is 0.2%, whereas 12% of those proteins in *Neisseria* meningitidis -derived OMVs (Lieberman, 2022). So far, the protein assay is still the prioritized method of OMVs quantification, such as Bradford and bicinchoninic acid (BCA) (Bitto et al., 2021). The heterogeneity of OMVs may significantly affect their protein content, indicating that the protein concentration may not accurately reflect the OMVs’ quantity. In addition, OMVs are not uniformly sized vesicles. Their size can also influence their cargo content and the mechanisms of their entry into host cells (Turner et al., 2018).

**TABLE 2:** The quantitative comparison of size, number, protein, BMV-associated DNA, and RNA of *S. aureus*, *P. aeruginosa*, and *H. pylori* strains.

Quantitative index	Quantitative methods	Quantitative results
Size (nm)	NanoSight NTA	*H. pylori* 26,695 < *S. aureus* < *P. aeruginosa* < *H. pylori* 251
Number (per CFU)	NanoSight NTA	*S. aureus* < *P. aeruginosa* < *H. pylori* 26,695 < *H. pylori* 251
Protein amount (μg/10^10^OMVs)	Qubit protein assay	*H. pylori* 26,695 < *H. pylori* 251 < *P. aeruginosa* < *S. aureus*
DNA amount (μg/10^10^OMVs)	Qubit high sensitivity DNA assay	*H. pylori* 251 < *H. pylori* 26,695 < *P. aeruginosa* < *S. aureus*

### Isolation and purification

The existing isolation and purification technologies of OMVs include ultracentrifugation, ultrafiltration, protein precipitation, and affinity isolation. Finally, harvested OMVs need to be incubated on agar growth plates to ensure no bacteria are present in the OMVs (Collins et al., 2021; Michel and Gaborski, 2022). Li and others have a summary of methods used to isolate OMVs in their published review, including the isolation principle, advantages, and disadvantages (see reference Li et al., 2020). Different isolation and purification technologies have advantages and limitations, so they are hard to meet the production demand of pure and specific OMVs. In addition, there is still no standard for isolating OMVs, and researchers often choose the isolation and purification methods based on their research purposes and experimental conditions. But existing each technique may alter the properties of isolated OMVs, so new reliable methods are needed to be developed to yield OMVs with desired purity and quantity.

### Drug-loading methods

The drug loading methods determine the PSs loading efficiency and membrane integrity of OMVs. There are usually two methods for drug loading in OMVs. The first method is to load drugs during the biogenesis process of OMVs via genetic engineering to modify parent bacteria or add drugs in the bacterial culture medium, leading to bacteria releasing the drug-loaded OMVs. The detailed mechanisms of biogenesis and “cargo” selectivity of OMVs are still unclear. So the drug loading efficiency of this method still needs further research and evaluation. The second method is OMV engineering which is based on the lipid bilayer structure of OMVs to load hydrophilic or hydrophobic compounds. Hydrophobic PSs can be passively loaded into isolated OMVs through co-incubation without any active substances. Hydrophilic PSs can penetrate into isolated OMVs through ultrasound, electroporation, or extrusion. There are many methods to encapsulate drugs, but the drug encapsulation efficiency is unsatisfactory (Ding et al., 2022). And some drug-loading methods may affect the size, charge potential, membrane rigidity, and integrity of OMVs, resulting in the endocytosis of any delivered drugs by target cells will be potentially altered.

## Conclusion and outlook

PDT has become an effective strategy for tumor treatment due to its unique action mechanism and low systemic toxicity. PDT has been clinically applied in treating superficial tumors and precancerous lesions with better therapeutic effects and application prospects. However, some limitations of PDT limit its therapeutic efficacy and widespread clinical application in deep-seated tumors. In recent years, researchers have made significant progress in the design of PSs, which has progressed from the first and second generations to the third generation, improving many shortcomings of traditional PSs. With the help of drug delivery carriers, PSs can achieve tumor targeting, TME/stimuli-response, and synergistic treatment of photodynamic/photothermal/chemo-/immuno-/gene therapy, providing new hope for precise and efficient tumor treatment. Existing research has confirmed that OMVs can also serve as delivery carriers like human EVs, and their unique immunogenicity contributes to they can be used as immune adjuvants to enhance the immune effect induced by PDT. OMVs can provide some positive benefits to photodynamic anticancer therapy and open a new avenue for designing versatile drug delivery carriers. We have realized that OMVs will have broad application prospects in photodynamic anticancer therapy. But many challenges need to be overcome, such as finding a way to balance detoxification and retaining enough efficacy of adjuvanticity, optimizing the purification method of OMVs, increasing production, and improving drug loading efficiency. Fortunately, some attempts have been made that may provide positive references. For instance, calcium phosphate (CaP) shells were employed to cover the surface of OMVs to attenuate toxicity (Qing et al., 2020). OMVs fused with cancer cell membranes to form hybrid OMVs have good homing and immune activation abilities (Wang et al., 2020). Therefore, we are confident that the deepening understanding of OMVs and the continuous progress of scientific research technology will contribute to the clinical transformation of OMVs as versatile drug delivery carriers in photodynamic anticancer therapy.
